# A de novo *TINF2*, R282C Mutation in a Case of Dyskeratosis Congenital Founded by Next-Generation Sequencing

**DOI:** 10.52547/ibj.3783

**Published:** 2022-10-28

**Authors:** Motahareh Khakzad, Zahra Shahbazi, Majid Naderi, Morteza Karimipoor

**Affiliations:** 1 Molecular Medicine Department, Biotechnology Research Center, Pasteur Institute of Iran, Tehran, Iran;; 2 Pediatric Cell and Gene Therapy Research Center, Gene, Cell & Tissue Research Institute, Tehran University of Medical Sciences, Tehran, Iran;; 3 Ali Ebne Abitaleb Hospital, School of Medicine, University of Medical Sciences, Zahedan, Iran

**Keywords:** Dyskeratosis congenita, Exome sequencing, Missense mutation, TINF2

## Abstract

**Background:**

Dyskeratosis congenita, an inherited and rare disease prevalent in males, is clinically manifested by reticulate hyperpigmentation, nail dystrophy, and leukoplakia. DC is associated with the increased risk of malignancy and other potentially lethal complications such as bone marrow failure, as well as lung and liver diseases. Mutations in 19 genes were found to be correlated with DC. Herein, we report a 12-year-old boy carrying a de novo mutation in *TINF2* gene.

**Methods:**

WES was performed on DNA sample of the proband, and the variant was investigated in the family by Sanger sequencing. Population and bioinformatics analysis were performed.

**Results:**

The NM_ 001099274.3(*TINF2*): c.844C>T (p.Arg282Cys) mutation was found by WES.

**Conclusion:**

There was no history of the disease in the family, and the variant was classified as a de novo mutation.

## INTRODUCTION

Dyskeratosis congenita is a rare inherited disease clinically characterized by a classic diagnostic triad of reticular skin pigmentation, nail dystrophy, and mucosal leukoplakia. Bone marrow failure or aplastic anemia is known to be prevalent in DC patients and is the most common cause of death in these cases^[^^1^^,^^2^^]^. Patients with DC are also susceptible to hematological malignancies and other solid tumors^[^^2^^]^. The age of onset is variable, and disease presentation can be mild to severe. Approximately 1/1,000,000 individuals have been inflicted by classic DC^[^^3^^]^. What makes the diagnosis of this disease difficult is its wide range of symptoms and varying presentation based on the clinical features^[^^4^^]^. DC is genetically diverse with autosomal recessive, autosomal dominant, and X-linked inheritance patterns. 

**Fig. 1:**
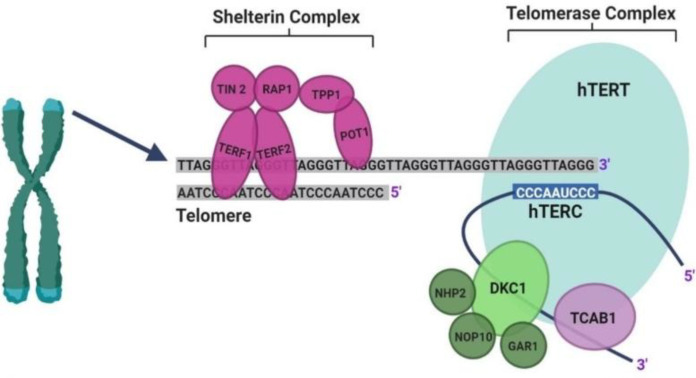
Scheme of telomerase and shelterin complex. Telomerase complex consists of the hTERT, RNA component (hTERC), and dyskerin protein complex (DKC1, NOP10, NHP2, GAR1, and TCAB1). TERT adds new telomeres (TTAGGG repeats) onto the chromosome end by using the template provided by TERC. The shelterin complex consists of six proteins (TRF1, TRF2, RAP1, POT1, TPP1, and TINF2) and protects telomeres and regulates telomerase^[^^9^^]^

DC is a telomere biology disorder^[^^5^^]^ in which synthesizing and maintaining telomeric DNA are performed by telomerase, a telomere-specific reverse transcriptase that uses a small region of its RNA subunit as a template to maintain the terminal sections of DNA in chromosomes^[^^6^^]^. The telomerase structure is comprised of the TERT, RNA component (TERC), and dyskerin protein complex (dyskerin, NOP10, NHP2, and GAR1)^[^^7^^]^. Besides the telomerase, the structure of shelterin helps form a stable telomere "cap". The structure of shelterin is comprised of six proteins, including TRF1, TRF2, RAP1, POT1, TPP1, and TIN2 (Fig. 1). This complex acts as a protector of telomeres and regulator of telomerase^[^^8^^]^. Dysfunctional telomeres are caused either by the gradual erosion of telomere as the result of the impaired replication of telomere ends or by the removal of the complex proteins of telomerase and shelterin^[^^9^^]^. Eleven genes (*DKC1*,* TERC*,* TERT*,* TINF2*,* NOP10*,* NHP2*,* WRAP53*,* ACD*,* RTEL1*,* PARN*, and* CTC1*) encoding the critical telomere components have been proved to be mutated in individuals with DC in different subtypes^[^^9^^]^.

According to the DC data registered in London, the major subtypes of disease are due to variants in *DKC1*, *TINF2*, *TERC*, and *TERT* in which hemizygous *DKC1* (dyskerin) variants are inherited in X-linked. Heterozygous *TIN2* and *TERC* variants are found with autosomal dominant inheritance pattern. Biallelic *NOP10*,* NHP2*,* WRAP53*,* PARN*, and *CTC1* variants are observed in autosomal recessive form. Heterozygous *TERT* and biallelic *RTEL1* and *ACD* variants that have been reported in both autosomal dominant and autosomal recessive patterns^[^^8^^]^. *TINF2* plays a key role in the assembly of the shelterin complex and its function. It connects the double-stranded DNA-binding proteins, TRF1 (TERF1) and TRF2 (TERF2), to the single-stranded DNA-binding unit TPP1/POT1^[^^10^^]^. Based on the previous studies, TIN2 mutation leads to defective targeting of telomerase to telomere ends^[^^11^^,^^12^^]^. 

In the present study, we report a DC patient with a de novo mutation that was detected by the WES method. Mutation confirmation and segregation analysis were performed in the proband and parents. To detect the prevalence of this variation in the Iranian population, we performed population study.

## MATERIALS AND METHODS

**Subjects**

The patient was a 12-year-old boy with normal parents of a Fars ethnic background. His clinical findings were as follows: abnormal nail, reticular skin pigmentation, leukoplakia, and hematologic abnormalities. An evaluation sheet was used to summarize the demographic information, including gender, date of birth, age of the onset of symptoms, clinical symptoms, age at diagnosis, family history and consanguinity, along with laboratory and molecular data. Blood samples were obtained from the patient and his parents, as well as from 100 control individuals. DNA was extracted from the whole blood using the salting out method. The quality of DNA was measured by a NanoDrop-2000-spectrophotometer (BioTek, USA).

**Genetic evaluation and confirmatory sequencing**

For identifying the genetic cause of the disease in the proband, WES was performed by Centogene, Germany. Briefly, the DNA library was prepared using an Agilent SureSelect Target Enrichment Kit preparation guide (Sure Select V6-Post kit, Santa Clara, CA, USA). The libraries were sequenced with Illumina HiSeq 2000/2500 platform. The WES variants list was annotated by Annovar and filtered in DC-associated genes (*ACD*,* CTC1*, *DKC1*,* NHP2*,* NOP10*,* PARN*,* RTEL1*,* TERC*,* TERT*, *TINF2*, and* WRAP53*). Synonymous variants and variants with a minor allele frequency of more than 1% in NHLBI exome sequence data (http://evs.gs. washington.edu/EVS/) and 1000 Genome project (http://www.1000genomes.org) were excluded. Effects of the identified variants were assessed by in silico prediction tools, including SIFT (https://sift.bii. a-star.edu.sg), PolyPhen-2 (http://genetics.bwh. harvard. edu/pph2), Combined Annotation Dependent Depletion (CADD), MutationTaster, and VarSome (https:// varsome.com), PredictSNP, MAPP, and PhD-SNP. The pathogenicity of the identified disease-attributable gene variants was re-evaluated using the updated guideline for the interpretation of molecular sequencing by the ACMG. In order to predict the effect of the identified variation, we used PhD secondary structure prediction server (https://npsa-prabi.ibcp.fr/ ) to calculate the overall effect of the amino acid variant on the secondary structure of the protein.

**Validation of results by ARMS-PCR and Sanger sequencing**

**Fig. 2:**
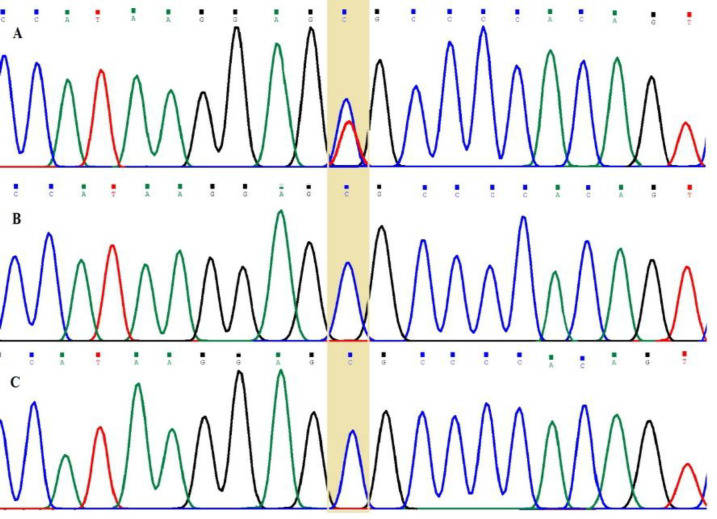
Sanger sequencing results showing the heterozygote state for the patient (A) and wild-type allele, homozygote state for his father (B) and his mother (C)

We confirmed the identified variant in the family by Sanger sequencing (Fig. 2). The frequency of the identified variant in the target gene was investigated in 100 normal individuals of the same ethnic group using ARMS-PCR.

## RESULTS

**Whole exome sequencing**

The patient was a 12-year-old boy with no family history of the disease. The hematologic examination showed pancytopenia, and aplastic anemia was suggested for him. WES was performed on DNA extracted from peripheral blood leukocytes. After bioinformatics analysis of the WES data, a heterozygous missense mutation, NM_ 001099274.3 (*TINF2*): c.844C>T (p.Arg282Cys), was found in the patient’s blood sample. The variant was located in the *TINF2* mutation cluster, which is the mutation hotspot where most of the variants identified so far are located. This variant was reported in ClinVar (https://www. ncbi.nlm.nih.gov/clinvar/), HGMD (https://www. hgmd.cf.ac.uk), and VarSome (https://varsome.com) databases. 

**Confirmation of WES results by Sanger sequencing and ARMS-PCR**

By using Sanger sequencing, we confirmed the c.844C>T (p.Arg282Cys) variant of *TINF2* gene in the heterozygous state in the studied patient, while his parents were homozygous for wild-type allele. Furthermore, the ARMS-PCR revealed the heterozygote state in the patient and the wild-type allele homozygote state in his parents. The Sanger sequencing and ARMS-PCR analyses showed the wild-type allele in homozygote state in all members of the studied normal population (Table 1).

**Table 1:** Allele frequency in probonds’ family and normal population

**Sample name**	**Number**	**Analyzed allele number**	**Genotype**
Patient	1	2	CT
Father	1	2	CC
Mother	1	2	CC
Control population	100	200	CC

***In silico*** **interpretation of identified variation**

**Fig. 3:**
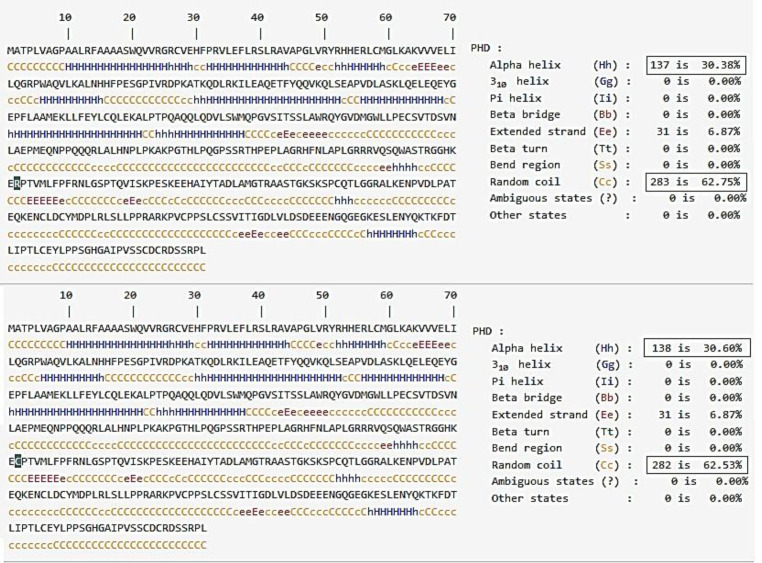
Secondary structure statistics in wild-type (up) and mutant type (down) proteins. The numbers changed is shown in a rectangular shape

The results of *in silico* analyzes confirmed the pathogenicity of *TINF2*, c.844C>T (p.Arg282Cys) variant. This variant was not reported on Iranome (http://www.iranome.ir) and has been categorized as the pathogenic variant based on the ACMG guideline (PS1, PS2, PS3, and PS4). The results of TINF2 protein alignment in 99 different species indicated that arginine 282 is located in a consensus region of TINF2 protein, which is next to a non-conserved region. Figure 3 depicts the secondary structures of the wild type and mutant TINF2 proteins. As shown in Figure 3, this single amino acid change led to slightly decreased random coil structure, while the alpha helix structure increased.

## DISCUSSION

DC is known as an inherited bone marrow failure syndrome characterized by mucocutaneous pigmentation and a wide range of other somatic abnormalities. This disease is genetically and clinically heterogeneous. An analysis of the genetic origin of DC has shown that a number of genes are the causative agent of the disease. All of these genes encode proteins that maintain telomere, either as part of telomerase or as part of the shelterin complex, which is responsible for capping and protecting telomeres. 

In the current study, we report a common *TINF2* mutation (R282C) associated with DC, which happened in a de novo manner. This variation was not observed in the patient’s parents. The results of different studies have pointed out the effect of this variation on the aggregation of telomeric protein in this complex and functional structure^[^^8^^]^. Some studies have also found de novo changes in some genes, which is believed to be responsible for DC. In the Knight et al.’s^[^^13^^] ^investigation, de novo variations were observed in the dyskerin (DKC1) gene in 8 out of 21 studied cases. Gene mutations causing human disease often help find protein functions previously unrecognized. This claim may be true about *TIN2*. *TINF2*, the gene encoding TIN2, is known as the second most commonly mutated gene in DC^[^^8^^]^. Being central to the shelterin complex, TIN2 links the telomeric proteins TRF1 and TRF2 to TPP1/POT1. The *TINF2* mutations found in DC caused very short telomeres^[^^14^^]^. DC-associated *TINF2* mutations are mainly de novo; however, they lead to drastically short telomeres in a single generation^[^^15^^]^. Conversely, TERT and TERC mutations are often inherited in an autosomal dominant pattern and induce progressive reduction of telomere length. Therefore, mutations in TERT and TERC genes cause more severe symptoms of the disease and can also affect several body systems during successive generations^[^^16^^]^. There is still a need to explain the basis for this rapid telomere shortening. It seems that all the mutations identified in the *TINF2* gene, which led to a significant decrease in the length of telomeres, are located in the central region of the protein. This region is called the DC cluster, has 30 amino acids length, and several mutation types, including missense, nonsense, and frameshift, have been reported in this domain^[^^17^^,^^18^^]^. While the most N-terminal truncations were confirmed to reduce the binding of TIN2S to TRF1, there is no evidence of the effect of these mutations on the interaction of TIN2S with TRF1, TRF2, or TPP1^[^^19^^,^^20^^]^. Accordingly, it has been supposed that the TIN2 mutations could affect other interactions^[^^14^^]^. Furthermore, while DC-associated *TINF2* mutations have been reported not to affect the overall telomerase activity, they reduce telomerase activity immuneprecipitated with TIN2S^[^^11^^]^. Earlier investigations have been mainly concerned with the shorter isoform of TIN2, but the data provided by Nelson et al.^[^^14^^]^ have demonstrated differences in the interactions of TIN2S and TIN2L with TRF1 and TRF2. Previous studies have reported that DC-associated mutations do not have a uniform effect on the interaction of TIN2S with TRF1, TRF2, and TPP1. Hence, it seems that mutations in mutation cluster domain of TIN2 change the composition of shelterin complex that leads to defect in telomere maintenance and protection^[^^19^^,^^20^^]^. Nelson et al.’s^[^^14^^]^ study has shown that arginine 282 plays a key role in the regulation of the interaction between TIN2L and TRF2 and its effect in TRF2. 

Some studies have found an interaction between TIN2L and TRF2 through F120. This is an important residue within TRFH domain of TRF2 known as a critical factor for the binding of TRF2 to Apollo and SLX4^[^^14^^,^^21^^]^. There may be a competition between TIN2L and Apollo and SLX4 proteins for binding to TRF2 via the TRFH domain. Therefore, in patients who have the R282H mutation, the reduced interaction between TIN2L and TRF2 may decline TIN2L binding to TRF2 at the telomere, allowing an increase in recruitment of Apollo, SLX4, or other factors that likely contribute to telomere shortening. TIN2S and TIN2L have a similar interaction with TPP1, which is not influenced by either R282H or S396A. However, researchers have found very specific results concerning the interactions of TIN2S and TIN2L with TRF1 and TRF2, as well as the very specific effects of R282H or S396A on TIN2S and TIN2L interactions^[^^14^^,^^20^^,^^22^^]^. These findings reveal the great influence of the most common DC-associated *TINF2* mutation on the ability of TIN2L, rather than TIN2S, to interact with the shelterin complex protein members

Telomeres play an important role in protecting the ends of chromosomes. Due to the surprising effects of very small changes in DNA, if the ends of chromosomes remain unprotected, the life of the cell and organism will be in danger. Various proteins and factors play key roles in creating the complex structure of the telomere. Mutations in the genes can lead to defects in the structure and function of telomeres. In the present study, de novo R282C mutation in *TINF2* gene was reported in a Dyskeratosis Congenital patient, and various studies and evidences about the effect of this variant on telomere function were investigated. However, these studies have not focused well on the longer TIN2 isoforms. Therefore, more studies are needed. 

## DECLARATIONS

### Ethical statement

Above-mentioned sampling protocols were approved by Pasteur Institute of Iran, Tehran, Iran (ethical code: IR.PII.AEC.1401.003). Informed consent was obtained for performing the studies from the patient’s parents and all volunteer participants.

### Data availability

The analyzed data sets generated during the study are available from the corresponding author on reasonable request.

### Author contributions

MK: performed molecular studies; ZS: analyzed the data and drafted the manuscript; MN: provided the medical history and clinical data; MK: designed and supervised the study and edited the manuscript.

### Conflict of interest

None declared.

### Funding/support

There is no funding supported this project.
